# Ultrasound in Skin Cancer: Why, How, and When to Use It?

**DOI:** 10.3390/cancers16193301

**Published:** 2024-09-27

**Authors:** Ximena Wortsman

**Affiliations:** 1 Department of Dermatology, Faculty of Medicine, Universidad de Chile, Lo Fontecilla 201 of 734 Las Condes, Santiago 8330111, Chile; xworts@yahoo.com; Tel.: +56-222446058; 2 Department of Dermatology, School of Medicine, Pontificia Universidad Catolica de Chile, Santiago 8331150, Chile; 3 Institute for Diagnostic, Imaging and Research of the Skin and Soft Tissues (IDIEP), Lo Fontecilla 201 of 734 Las Condes, Santiago 7591018, Chile; 4 Department of Dermatology and Cutaneous Surgery, Miller School of Medicine, University of Miami, Miami, FL 33146, USA

**Keywords:** skin cancer, ultrasound, basal cell carcinoma, squamous cell carcinoma, melanoma, dermatofibrosarcoma protuberans, lymphoma, angiosarcoma, liposarcoma, locoregional staging, dermatology

## Abstract

**Simple Summary:**

Skin cancer is a major global problem, and over the last decade, ultrasound technology has advanced significantly, enhancing our ability to detect and identify the most common types of skin cancer, including both the primary tumor and its locoregional metastases.

**Abstract:**

Background: Skin cancer is the most common cancer in human beings. Ultrasound is a powerful and non-invasive imaging technique that has expanded its use in dermatology, including in the skin cancer field. The full range of critical anatomical information provided by ultrasound cannot be deduced from a naked eye examination, palpation, or other imaging techniques such as dermoscopy, confocal microscopy, magnetic resonance imaging, or PET-CT (Positron Emission Tomography-Computed Tomography). Methods: This review practically analyzes the main ultrasonographic features of the most common types of skin cancers and the performance of the locoregional staging according to the literature, which is illustrated by state-of-the-art clinical and ultrasonographic correlations. Results: The most common types of skin cancer show recognizable ultrasonographic patterns. Conclusions: Among the current radiological imaging techniques, ultrasound has the highest axial spatial resolution. Compared to other imaging techniques used in dermatology, it shows the great advantage of penetrating the soft tissues thoroughly, which allows us to detect and identify the most common skin types of skin cancer, including both the primary tumor and its locoregional metastases.

## 1. Introduction

Skin cancer is the most common cancer in human beings, and so far, its imaging studies in dermatology have been mostly focused on dermoscopy and confocal microscopy [1,2,3,4].

On the other hand, ultrasound (US) is a non-invasive imaging technique based on non-audible frequencies that has been growing exponentially in the field of dermatology. The most common US applications include benign cutaneous tumors and pseudotumors, skin cancer, vascular anomalies, inflammatory cutaneous conditions, nail pathologies, and esthetic complications [5,6,7].

Technological advances in US have increased the axial spatial resolution from 100 µm to 30 µm through the development of much higher frequencies up to 71 MHz, which provide images comparable with the lower magnification of histology. It is essential to keep in mind the limitations of dermatologic ultrasound, which are the lesions that measure less than 0.1 mm at 15 MHz, the lesions that measure less than 0.03 mm at 70 MHz, the only epidermal lesions (in situ), and the detection of pigments such as melanin. Thus, these limitations seem reasonable to detect tumors that involve the dermis and beyond [8].

The axial spatial resolution of high- and ultra-high-frequency ultrasound at 15 to 71 MHz is much higher than that of magnetic resonance imaging (MRI) at 3.0 Tesla (T), computed tomography (CT), and positron emission tomography (PET-CT). MRI axial spatial resolution at 3.0 T, the most commonly used device, is 400 µm. In fact, lesions measuring less than 3 mm on 3.0 T MRI may not be detected or characterized. PET-CT has issues detecting or characterizing lesions that measure less than 8 mm. In contrast, US detects submillimeter lesions. The indications of MRI, CT, and PET-CT are the staging, not the study of the primary skin tumor.

Optical Coherence Tomography (OCT) offers higher axial spatial resolution than ultrasound (10 μm); however, it has limited penetration because it can only observe up to 2 mm depth. While this may be enough for detecting superficial skin cancer, it poses an issue for measuring deeper lesions, which is critical in cutaneous cancer [7].

US has powerful advantages over other imaging techniques, such as the possibility to detect blood flow in real time through the color or power Doppler application without the need to inject a contrast media. Another important benefit of US is the ability to penetrate the tissues without losing resolution. The latter capacity of ultrasound is due to the development of multifrequency probes that can cover wide ranges of depth [5,9,10]. The US penetration ability is critical in skin cancer, where it is important not to miss a tumor that infiltrates deeper layers [11].

Therefore, in skin cancer, to have an imaging modality that provides information in advance of the primary tumor, staging, and vascularity, including the relevant vessels in the periphery of the tumor, is quite an important advancement [11].

This is even more relevant in skin cancers affecting the facial region, where the dermis is thinner compared to other corporal regions, and the possibility of involving deeper layers such as cartilage, muscle, or bone is higher, particularly for the high-risk of recurrence subtypes [7,12,13,14,15].

Ultrasound can support the decision about the type of surgery performed (standard or Mohs); therefore, the time spent in the operating room can be programmed reasonably [7,11,14]. Moreover, Mohs surgery has a high percentage of good oncologic results, but recurrences have been reported in 2 to 3% of patients who undergo this treatment [16]. Thus, recurrences after Mohs surgery have been reported, which could be a multifactor issue that could be more critical in deep or centrofacial skin cancers. Consequently, US can support anatomical information that could be relevant for surgical planning [11,12,13,14,15,17].

When performing ultrasounds in dermatology, there are requisites that include a color Doppler ultrasound machine with a linear or compact linear probe working with a frequency equal to or higher than 15 MHz and an operator trained in imaging and dermatologic conditions [6,7,9,10,18,19].

The higher the frequency, the higher the axial spatial resolution [8]; however, it is essential to consider the range that probes present. This allows us to adjust the depth in the screen to properly see the target.

US can detect the primary tumor and also support a locoregional staging that is focused on the detection of satellite (<2 cm from the primary tumor), in transit (≥2 cm from the primary tumor), and nodal metastases [6,7,11,12,13,14,15].

In this review, we analyze the role of ultrasound in the most common types of skin cancers.

### 1.1. Ultrasound Protocol

According to the literature and the published guidelines for performing dermatologic ultrasound examinations, the operator should apply a copious amount of gel on top of the cutaneous lesion and then sweep the area in greyscale and color Doppler in at least two perpendicular axes. Spectral curve analysis (pulsed Doppler) is used to detect the type (arterial or venous), thickness, and velocity of the blood flow (cm/s) within and in the periphery of the tumor [9,10,18].

The primary tumor or its recurrence or remnant areas are measured on all axes (transverse, thickness, and longitudinal axes) in mm or cm according to the tumor size [6,7,11,14,15,20].

Any involvement of deeper layers, including cartilage, muscle, or bone, is reported. Additionally, the relevant vessels close to the tumor, such as the main arteries, are mentioned [6,7,11,12,13,14,15,20,21].

The locoregional staging starts at the primary tumor or scar site and follows the lymphatic drainage regions up to the corresponding nodal stations [6,7,11,12,13,14,15,17,20,21].

The illustrative cases presented in this review were performed with two ultrasound machines. The first machine was a Logiq E10 (General Electric Health Systems, Waukesha, WI, USA), equipped with a compact linear probe that presents an upper frequency of 24 MHz. The second device was a Vevo MD (VisualSonics Fujifilm, Toronto, CA, USA), with a linear probe that presents an upper range of 71 MHz.

The figures presented in this review belong to patients who signed an informed consent form pertaining to the publication of their images, and all the examinations were performed following the Helsinki principles of medical ethics.

### 1.2. Ultrasound Patterns of Skin Cancer

US has been reported to be useful in common types of skin cancer. For this purpose, we will categorize the malignant lesions and analyze the ultrasonographic features of each tumor (Table 1).

### 1.3. Keratinocytic Tumors (Non-Melanoma)

These are the most frequent malignant cutaneous tumors in human beings, and their category has been renamed to keratinocytic tumors (non-melanoma). They can be divided into basal cell carcinoma (BCC), the most common type, and squamous cell carcinoma (SCC).

#### 1.3.1. Basal Cell Carcinoma (BCC)

##### Primary BCC Tumor

This type of skin cancer presents several subtypes that can be divided into low-recurrence and high-recurrence histologic risk categories. The main representatives of low recurrence risk are the nodular and adenoid-cystic types. the types at high risk of recurrence are the micronodular, morpheiform, metatypical, infiltrative, and sclerosing subtypes, among other subtypes [22].

On ultrasound, BCC is shown as a hypoechoic lesion, often an oval, rounded shape, or with a ribbon, elongated, or rosary bed pattern that presents irregular borders and located in the dermis and sometimes deeper layers. Observing anechoic lacunar cystic spaces within the lesion is possible in the adenoid-cystic type of BCC [6,12,15,20,22,23,24,25,26,27,28,29,30].

One of the pathognomonic signs of BCCs is the presence of inner hyperechoic spots that are a marker of the degree of aggressiveness. These hyperechoic spots are not reported in squamous cell carcinoma or melanoma [6,12,15,20,22,23,24,25,26,27,28,29,30,31].

For example, high-risk recurrence subtypes tend to demonstrate a higher number of hyperechoic spots. A cut-off of >7 hyperechoic spots within the tumor at 15 MHz has been reported as significant for discriminating high-risk versus low-risk recurrence subtypes [32].

In superficial variants of BCCs, these hyperechoic spots can be less visible or absent. In adenoid-cystic variants, anechoic spaces may be seen within the tumor.

The presence of mixed high- and low-recurrence-risk subtypes of BCC can be detected on ultrasound and show different areas with high and low numbers of hyperechoic spots in the same lesion [6,11,14,15,32].

A high correlation of the thickness of BCCs between ultrasound and histology has been described in the literature. In a systematic review, ultrasound has been noted to provide accurate depth measurements, especially for BCCs > 1 mm [23,24,33].

US has been reported as significant for discriminating the deep margin of BCCs with a sensitivity of 96%, specificity of 84%, and accuracy of 91% for measuring deep tumor margins [23]. An excellent correlation was found between the BCC thickness measured by ultra-high frequency (70 MHz) and histology (interclass correlation ≥ 0.80) [28,31].

Ultrasound has no issues observing clinically challenging locations of BCCs for other imaging modalities, such as the ear pinna, nose, lips, and eyelids [34].

**Figure 1:**
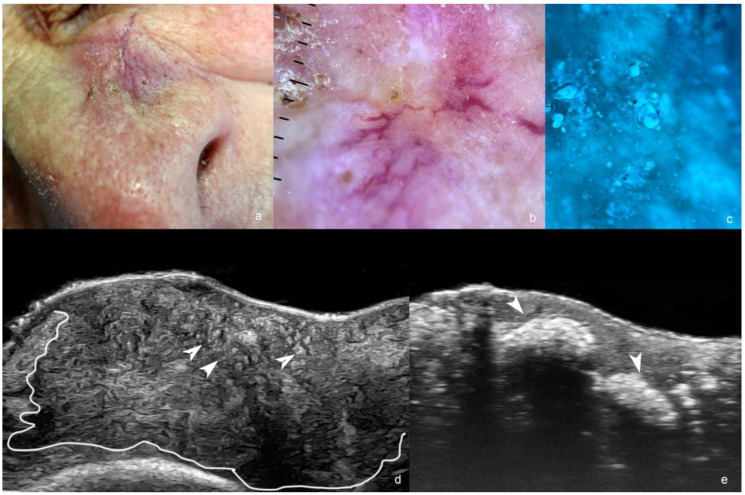
(**a**–**e**). Basal cell carcinoma. (**a**). Clinical photograph of the lesion in the paranasal region. (**b**). Dermoscopy. (**c**). Wood light of the lesion. (**d**,**e**). Ultrasound images ((**d**), greyscale at 24 MHz and (**e**), at 71 MHz) demonstrate an ill-defined hypoechoic dermal, hypodermal, and musculoaponeurotic mass (outlined) that presents multiple hyperechoic spots (some of them marked with arrowheads pointing up, as shown in (**d**) and calcifications pointing down, as shown in (**e**).

Ex vivo ultrasonographic analysis of BCCs has also been reported in the literature [35,36,37] (Figure 1).

##### Post Operative Recurrence or Remnant BCC

The usefulness of ultrasound in detecting a BCC´s recurrence is reported in the literature [17].

Nevertheless, the request for an ultrasound of the primary lesion is preferred over post-operative examination due to the potential anatomical distortion of the tissues. 

**Figure 2:**
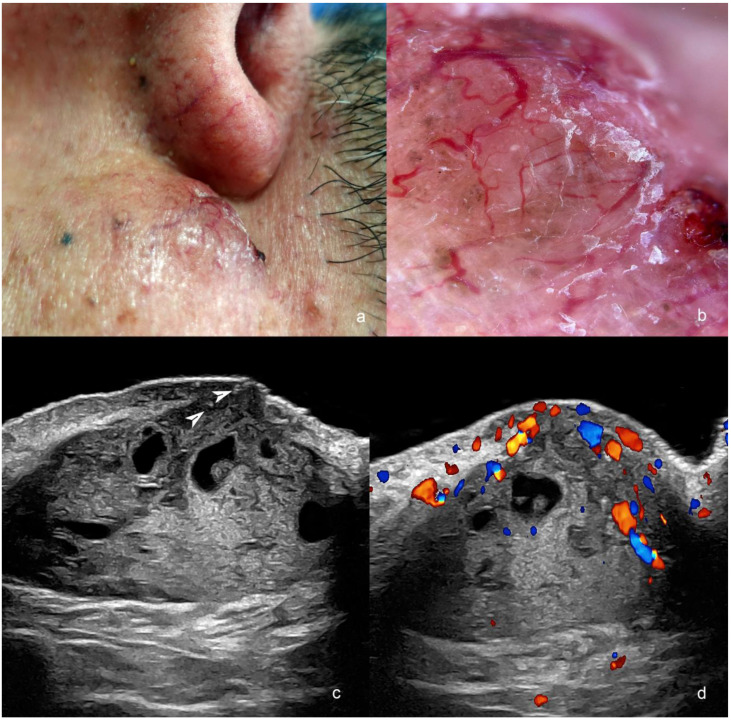
(**a**–**d**). Post-operative recurrence of basal cell carcinoma (post-Mohs surgery) in the medial aspect of the cheek adjacent to the nasofold line. (**a**). Clinical image. (**b**). Dermoscopy. (**c**,**d**). Ultrasound ((**c**), greyscale and (**d**), color Doppler, transverse views) present hypoechoic dermal and hypodermal mass with hyperechoic spots (arrowheads) and anechoic lacunar areas. The mass has increased blood flow on color Doppler ultrasound, mainly in the superficial part.

The recurrent or remnant tumor shows similar ultrasonographic morphology to the primary tumor, and typically, there are scarring signs or some inflammatory changes in the periphery that take the form of hypoechoic laminar or fibrillar structures, heterogenous echogenicity, and sometimes low hypervascularity. Occasionally, there are two or more foci of recurrence, usually in the original borders of the primary tumor [6,7,11,14,15,17] (Figure 2).

#### 1.3.2. Squamous Cell Carcinoma (SCC)

##### Primary SCC Tumor

There are two main types of SCC: intraepidermal SCC (Bowen’s disease) and the usual tumor that involves the dermis and beyond [38].

Since this type of tumor is more aggressive than BCC, using ultrasound to study SCC could help detect locoregional metastases besides the primary tumor [38,39].

On ultrasound, SCC appears as a hypoechoic lesion with irregular borders and no hyperechoic spots. In fact, the lack of hyperechoic spots in BCC has been reported as significant for discriminating between BCC and SCC [15]. Other SCC ultrasound features are reported, such as epidermal irregularities, a crumpled surface, epidermal detachment, ulceration, bulging, convex or concave, and flat shapes. SCC is commonly located in the dermis but could infiltrate deeper tissues such as subcutaneous, muscle, cartilage, or bone [15,25,39,40,41,42].

On color Doppler, SCC presents a higher vascularity than BCC with low-flow vessels [6,7,11,14].

Locoregional staging is commonly performed in SCC to detect satellites and in-transit or nodal metastases. Ultrasonographers have reported a sensitivity of 91% (95% CI, 71–99%) and a specificity of 78% (95% CI, 72–83%) for detecting metastases of SCC [43].

**Figure 3:**
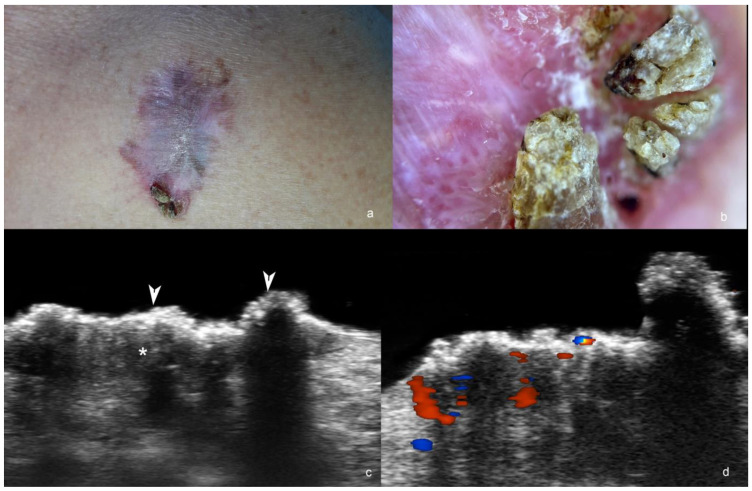
(**a**–**d**). Squamous cell carcinoma. (**a**). Clinical image of the lesion at the anterior aspect of the thorax. (**b**). Dermoscopy. (**c**,**d**) Ultrasound at 71 MHz ((**c**), greyscale; (**d**), color Doppler, transverse views) show ill-defined hypoechoic dermal structure (*) without hyperechoic spots and with crumpled, exophytic and irregular epidermis (arrowheads). On color Doppler (**d**), the lesion has increased vascularity.

Furthermore, radiologic imaging in high-stage SCC has been shown to influence management and appears to positively impact outcomes [39,41] (Figure 3).

### 1.4. Bowen Disease

Since this is the intraepidermal involvement of SCC, the detection is better when ultra-high frequency devices (≥50 MHz) are used. These show epidermal irregularities, a crumpled and wavy epidermal surface, and hypoechogenicity of the lower part of the epidermis. On color Doppler, in some cases, it is possible to detect hypervascularity with slow vessels in the underlying dermis [42,44].

### 1.5. Post-Operative Recurrence or Remnant SCC

**Figure 4:**
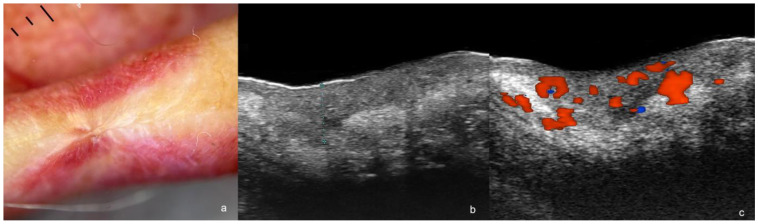
(**a**–**c**). Post-operative recurrence of squamous cell carcinoma. (**a**). Dermoscopy shows a lower lip scar. (**b**,**c**). Ultrasound images at 71 MHz ((**b**), greyscale and (**c**), color Doppler, transverse views) present a hypoechoic dermal and hypodermal structure (thickness marked in (**b**)) with irregular borders and increased vascularity (**c**).

The recurrent or remnant SCCs present ultrasonographic morphology similar to the primary tumors. Scarring areas with a laminar pattern and heterogenicity of the tissues can be detected in the periphery of the tumor(s). A variable degree of vascularity with low-flow vessels can be detected within or at the lesion’s periphery (s) [17,34,45] (Figure 4).

## 2. Melanoma

### 2.1. Primary Tumor

Melanoma is the most lethal type of skin cancer; however, it is the least common. The literature shows a high correlation of the thickness between ultrasound and histology. Moreover, a Breslow ultrasonographic index has been reported. The performance of an ultrasound examination in primary melanoma makes sense, since the prognosis and the decision of the sentinel lymph node procedure are influenced by the thickness of the tumor. If this information is known in advance, it could potentially avoid the need for a second surgery to extend the surgical margins.

On ultrasound, melanomas appear as hypoechoic dermal lesions that could easily extend to the deeper tissues. Frequently, they show a fusiform shape and lead to upward displacement of the epidermis. In the periphery of the tumor, there are inflammatory signs such as decreased dermal echogenicity and increased subcutaneous echogenicity. On color Doppler, melanomas are usually hypervascular with low-flow vessels. This is due to their angiogenic nature [6,12,13,14,15,33,39,46,47,48,49,50].

Literature reports that frequencies ≥70 MHz demonstrate a strong correlation with histopathology. Higher ultrasound accuracy has been seen for melanomas with Breslow depth >0.75 mm. Deeper melanomas can require probes with frequencies lower than 20 MHz. Minor variations in the reports of the thicknesses between ultrasound and histology may be due to inflammatory infiltrates, nevus adjacent to the lesion, and shrinkage during the pathological specimen preparation [6,12,13,14,15,33,39,46,47,48,49,50].

Ultrasonographic locoregional staging is essential in these cases to detect satellite, in-transit, and nodal metastases [6,14,15,21,33,51,52,53,54,55,56]. The axial spatial resolution of ultrasound is higher than that of MRI (magnetic resonance imaging) and PET-CT (positron emission tomography-computed tomography) [6,7]. Therefore, some locoregional metastases may not be detected in MRI or PET-CT but can be observed on ultrasound (false negatives) [57,58]. False positives in PET-CT are reported in patients with deposits of cosmetic fillers and focal sites of inflammation, among other causes, which can be supported in their diagnosis with ultrasound [58,59,60].

**Figure 5:**
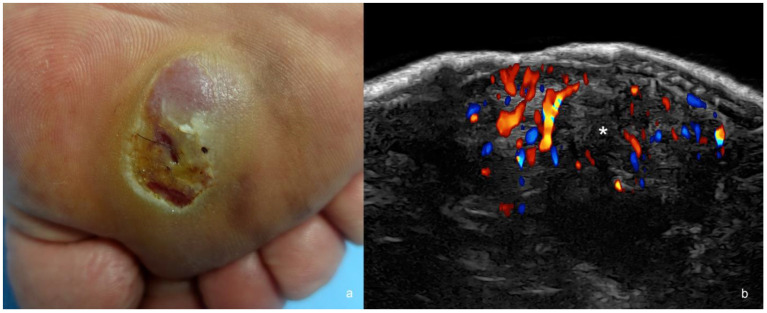
(**a**,**b**). Melanoma. (**a**). Clinical image in the plantar region. (**b**). Color Doppler ultrasound image at 24 MHz (transverse view) demonstrates hypoechoic dermal and hypodermal mass (*) with irregular borders and prominent hypervascularity.

Ultrasound-guided presurgical marking of melanomas, which can be performed with a surgical pen or tape, has been documented in the literature [35]. Ex vivo ultrasound of the in-transit melanoma metastases is also reported as useful [35] (Figure 5).

### 2.2. Locoregional Metastases

Satellite (<2 cm from the primary tumor or scar) and in-transit (≥2 cm from the primary tumor or scar) metastases appear on US as hypoechoic nodules, which are commonly located in the dermis and/or subcutaneous tissue, but most frequently found in the subcutaneous tissue. Increased echogenicity of the fatty subcutaneous tissue in the periphery of the nodules is common [12,13,21,34,51,61].

The “tail” sign (a thin hypoechoic prolongation from one or both poles of a superficial metastasis) and a “string” sign (multiple in-transit lesions connected to each other in a rosary image) have been reported in subcutaneous melanoma metastases [62].

**Figure 6:**
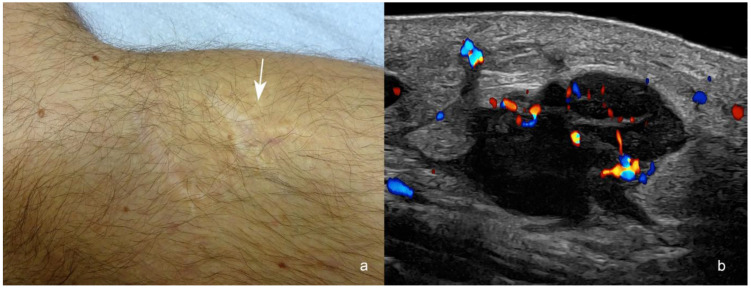
(**a**,**b**). Melanoma in-transit metastasis. (**a**). Clinical image shows the scar of the melanoma surgery and the location of a palpable pseudonodular structure in the posterior part of the medial aspect of the knee (arrow). The melanoma was originally located in the anterior part of the scar. (**b**). Color Doppler ultrasound at 24 MHz (longitudinal view) presents a hypoechoic hypodermal mass with spiculations and lobulations at 3 cm posteriorly from the primary tumor location (arrow in (**a**)). Notice the increased echogenicity of the adjacent hypodermis and the prominent vascularity within the mass. There is also a “tail sign” in the upper and lower borders of the nodule.

On color Doppler, increased vascularity with low-flow vessels is frequently observed within and in the periphery of the nodules [11,14,15] (Figure 6).

On an ultrasound, metastatic lymph nodes can present areas of asymmetrical cortical thickening, internal hypoechoic cortical or medullar nodules, a rounded shape, a transverse size > 1 cm, and loss of the cortex–medulla definition [6,7,11,14,15,56].

In melanoma, it is common to see anechoic areas within the infiltrated lymph nodes, which correspond to highly cellular regions and not to necrosis [53,63].

On color Doppler, the normal centripetal flow of the lymph nodes is replaced by a chaotic cortical flow with low-velocity vessels [11,12,13,14,15,21,51].

To date, high-resolution US cannot substitute the sentinel lymph node biopsy, mainly because of low sensitivity due to many missing micrometastases [64]. Nevertheless, ultrasound can guide cytology or biopsy [56,65]. In the future, the development of even higher ultra-high-frequency probes and photoacoustic devices may address this issue.

## 3. Dermatofibrosarcoma Protuberans (DFSP)

Dermatofibrosarcoma protuberans is a locally aggressive, superficial mesenchymal neoplasm with fibroblastic differentiation (fibrohistiocytic). The ultrasound’s usefulness in detecting the tumor and defining its borders has been reported, which may help in surgical planning [14,15,66,67].

Ultrasonographically, DFSP shows a heterogenous dermal and hypodermal structure with a hypoechoic or heterogeneous area in the upper or middle parts and a hyperechoic zone at the bottom that usually presents pseudopods. Besides, this tumor commonly displaces upward the epidermis. Some authors have described the pattern of DFSP as “jellyfish-like” [14,15,66,67,68,69].

In DFSP, the presence of tentacle-like projections or a “claw” sign in the primary tumor has been reported. In contrast, the literature mentions oval, lobulated, and irregularly shaped lesions in recurrent tumors [66,67].

**Figure 7:**
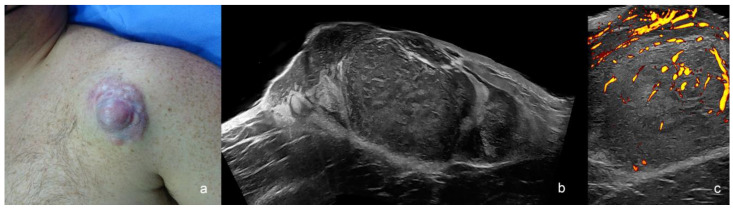
Dermatofibrosarcoma protuberans (**a**–**c**). (**a**) Clinical image. (**b**,**c**) Ultrasound images at 24 MHz ((**b**), greyscale panoramic view; (**c**). power Doppler; transverse views) present dermal and hypodermal mass with hypoechoic and hyperechoic areas that show pseudopods in the fatty tissue. On power Doppler, there is increased vascularity within the mass.

On color Doppler, the vascularity can be variable from mild to prominent with low-flow vessels [11,14,15] (Figure 7).

## 4. Merkel Cell Tumor

This malignant tumor is derived from Merkel cells. It presents as a hypoechoic focal area, nodule, or pseudonodule, commonly exophytic, with ill-defined borders and chaotic hypervascularity that presents low-velocity vessels.

**Figure 8:**
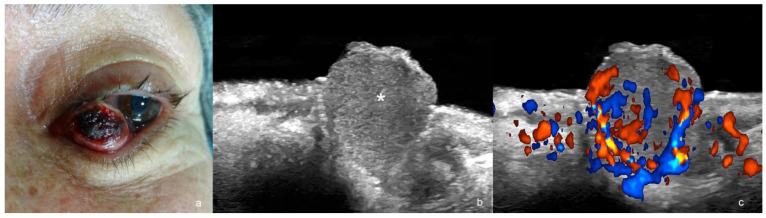
(**a**–**c**). Merkel cell carcinoma. (**a**). Clinical photograph. (**b**,**c**). Ultrasound images at 24 MHz ((**b**), greyscale and (**c**), color Doppler, longitudinal views) show exophytic hypoechoic mass (*) that involves the dermis, the orbicularis muscle of the lower eyelid, and the underlying intraorbital extraconal fat.

Ultrasound can support the detection of the lesion and definition of the borders [11,14,15,70,71] (Figure 8).

## 5. Liposarcoma

This is a fatty tissue malignant tumor. A liposarcoma is suspected when a > 5 cm, heterogenous echogenicity and hypervascular mass is detected in the subcutaneous tissue. However, myxoid variants of liposarcoma can be fully hypoechoic or contain multiple anechoic lacunar areas within the tumor.

**Figure 9:**
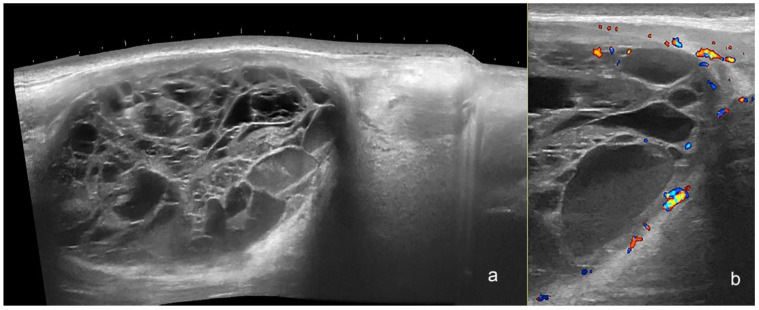
(**a**,**b**). Myxoid liposarcoma. Ultrasound images (**a**), panoramic greyscale longitudinal view; (**b**) color Doppler transverse view at the posterior aspect of the thigh presents an oval-shaped hypodermal mass with a mixed echogenicity and multiple anechoic lacunar spaces and hypoechoic areas. There is prominent hyperechogenicity compatible with edema in the adjacent hypodermis and a posterior acoustic reinforcement below the mass. On color Doppler, there is increased vascularity in the periphery of the structure.

The vascularity can be variable but is usually low-to-intermediate degree with low-flow vessels [11,72,73,74] (Figure 9).

## 6. Angiosarcoma

This malignancy represents a proliferative neoplastic growth of vessels. On ultrasound, it shows as a hypoechoic or heterogeneous subcutaneous structure with ill-defined borders and prominent hypervascularity [75].

The use of ultrasound has been reported for studying angiosarcoma post-radiotherapy and Kaposi sarcoma (KS) [75,76].

**Figure 10:**
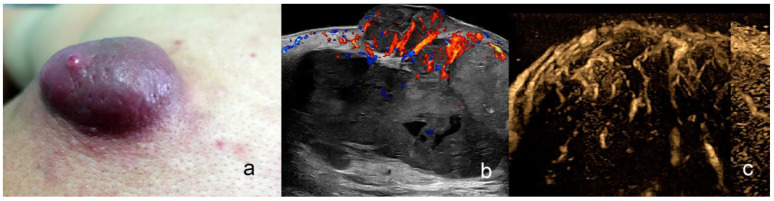
(**a**–**c**). Angiosarcoma. (**a**). Clinical photograph dorsal region. (**b**,**c**). Ultrasound images ((**b**), color Doppler, and (**c**), echoangio B-Flow) demonstrated dermal and hypodermal hypoechoic mass with some lacunar anechoic areas and prominent hypervascularity.

Commonly, KS has nodular and interstitial types of presentation. The nodular variant appears on ultrasound as a hypervascular hypoechoic nodular dermal and/or hypodermal structure. The interstitial form shows as an ill-defined hypoechoic and usually hypervascular dermal and/or subcutaneous area [11,75,76] (Figure 10).

## 7. Lymphomas

**Figure 11:**
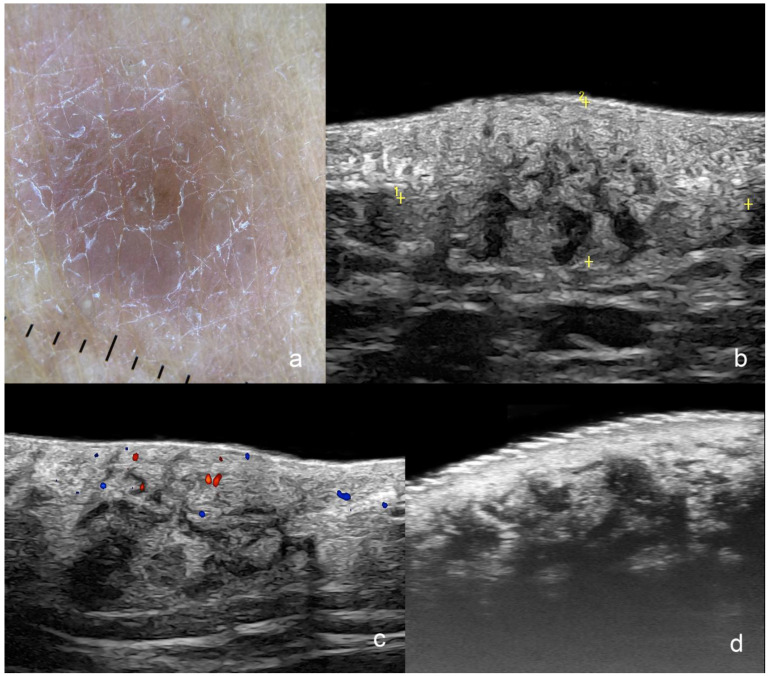
(**a**–**d**). Cutaneous Lymphoma. (**a**). Clinical dermoscopy image at the dorsal region. (**b**–**d**). Ultrasound images ((**b**,**c**) at 24 MHz; (**b**), greyscale and (**c**), color Doppler; longitudinal views; (**d**), greyscale at 71 MHz; longitudinal view) show a focal dermal and hypodermal infiltrative area with ill-defined borders (between markers) and heterogenous echogenicity that presents some hypoechoic disorganized bands and pseudonodular structures. On color Doppler (**c**), there is a mild increase in the regional vascularity.

Primary cutaneous lymphoma (PCL) is a rare form of extranodal non-Hodgkin’s lymphoma [15,77,78]. On ultrasound, hypoechoic nodules, pseudonodular lesions, or areas with dermal and/or hypodermal infiltration showing heterogeneous echogenicity have been reported. Thickening of the dermis without any calcifications or central necrosis has also been mentioned. Nodular and pseudonodular lesions have been described predominantly in B-cell lymphomas, and diffusely infiltrative lesions seem more common in T-cell lymphomas. These lesions can extend into the hypodermis. On color Doppler imaging, the lesions commonly show a variable degree of hypervascularity, frequently of low to intermediate level, with slow-flow vessels [15,77,78,79] (Figure 11).

## 8. Cutaneous Metastases

Several cancers can generate metastases to the skin, such as kidney, lung, stomach, pancreas, or breast, in addition to skin cancers [47,59,80,81,82,83].

**Figure 12:**
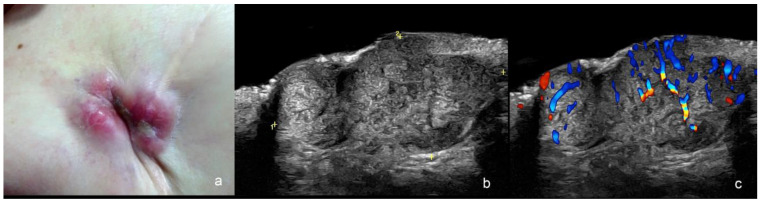
(**a**–**c**). Cutaneous metastasis of breast cancer in the scar of the mastectomy. (**a**). Clinical image. (**b**,**c**). Ultrasound images at 24 MHz ((**b**), greyscale and (**c**), color Doppler) present a hypoechoic dermal and hypodermal mass (between markers in (**b**)) with lobulated borders and hypervascularity on color Doppler (**c**).

On ultrasound, cutaneous metastases present as hypoechoic dermal and/or subcutaneous masses with lobulated and/or ill-defined borders. There is increased echogenicity of the surrounding subcutaneous tissue and hypervascularity with slow-flow vessels within and in the lesion’s periphery. These masses can be single or multiple, which can be detected on ultrasound (Figure 12).

## 9. Conclusions

Color Doppler US is a valuable imaging technique for a wide variety of skin cancers. It provides a full range of critical information on the primary tumor and its locoregional staging that cannot be deducted from a naked eye examination, palpation, dermoscopy, confocal microscopy, magnetic resonance imaging, or PET-CT.

Ultrasonography can be critical in some corporal regions, such as the face, where the skin is thin, and a deeper layer involvement is reasonably possible. 

This technique needs special equipment with high- and ultra-high-frequency probes according to the tumor size, a trained operator, and standardized protocols. In this setting, the results are usually correlated with histology.

In the future, an increased use of this technique with the incorporation of ultrasound into the management guidelines and the research trials in skin cancer appears to be sorely needed.

## Figures and Tables

**Table 1:** Ultrasonographic features of common types of skin cancer.

Type of Skin Cancer	Common Grayscale Ultrasonographic Signs	Vascularity on Color Doppler
Basal Cell Carcinoma	hypoechoic, hyperechoic spots, oval, round, elongated, rosary-bead shape, variants with anechoic spaces, irregular borders	low degree
Squamous Cell Carcinoma	hypoechoic, crumpled, wavy or irregular epidermis, convex, concave flat, bulging or fusiform shape, irregular borders	intermediate degree
Melanoma	hypoechoic, fusiform, flat or nodular, irregular or ill-defined borders	high degree
Dermatofibrosarcoma Protuberans	heterogeneous, hypoechoic on the surface and/or intermediate parts, and hyperechoic at the bottom with pseudopods or jellyfish-like, tentacle, or claw signs	intermediate degree
Merkel Cell Tumor	hypoechoic, nodule, pseudonodule, exophytic, ill-defined borders	high degree
Liposarcoma	hypoechoic, heterogeneous, myxoid variant with anechoic spaces, more than 5 cm	intermediate degree
Angiosarcoma	hypoechoic or heterogeneous, ill-defined borders; Kaposi may present a nodular shape	high degree
Lymphomas	hypoechoic or heterogeneous nodules and pseudonodules and ill-defined focal areas	low to intermediate degree
Cutaneous Metastases	hypoechoic with lobulated and irregular borders, single or multiple, increased echogenicity of the hypodermis in the periphery	intermediate to high degree
